# Study on Cr(VI) Leaching from Cement and Cement Composites

**DOI:** 10.3390/ijerph15040824

**Published:** 2018-04-22

**Authors:** Adriana Estokova, Lenka Palascakova, Maria Kanuchova

**Affiliations:** 1 Department of Material Engineering, Faculty of Civil Engineering, Institute of Environmental Engineering, Technical University of Kosice, Vysokoskolska 4, Kosice 04200, Slovakia; lenka.palascakova@gmail.com; 2 Faculty of Mining, Ecology, Process Control and Geotechnology, Institute of Montaneous Sciences and Environmental Protection, Technical University of Kosice, Park Komenskeho 19, Kosice 04384, Slovakia; maria.kanuchova@tuke.sk

**Keywords:** hexavalent chromium, cement, building materials, silica fume, zeolite

## Abstract

This paper reports an experimental study on hexavalent chromium leaching from cement samples and cement composites containing silica fume and zeolite additions that were subjected to various leaching agents. The water-soluble Cr(VI) concentrations in cements ranged from 0.2 to 3.2 mg/kg and represented only 1.8% of the total chromium content. The presence of chromium compounds with both chromium oxidation states of III and VI was detected in the cement samples by X-ray photoelectron spectroscopy (XPS). Leaching tests were performed in a Britton-Robinson buffer to simulate natural conditions and showed increased dissolution of Cr(VI) up to 6 mg/kg. The highest amount of leached hexavalent chromium was detected after leaching in HCl. The findings revealed that the leaching of chromium from cements was higher by 55–80% than that from the cement composites. A minimum concentration was observed for all cement samples when studying the relationship between the soluble Cr(VI) and the cement storage time.

## 1. Introduction

Chromium, one of the 25 most abundant elements in the Earth’s crust with an average concentration of 100 ppm [1], is an indelible trace element in raw materials for manufacturing cement clinker [2]. However, chromium in cements can originate not only from the raw materials (clays and limestone- or iron-based ingredients) but also from other sources, such as refractory bricks lining the kiln, mineral admixtures, and grinding media in the final finishing mills [3]. Among the various forms of chromium, Cr(III) is dominant in natural, raw materials [4]. The Cr(III) form is relatively nonreactive and less toxic; however, it can be oxidized into hexavalent species at high temperatures under an oxidizing atmosphere and alkaline conditions in cement kilns [5]. Cr(VI) is the most hazardous form of chromium because of its toxicity, solubility, and mobility. The hexavalent form of chromium is acidic, forming chromates [(CrO_4_)^2−^] and dichromates [(Cr_2_O_7_)^2−^]. The mutagenetic and carcinogenetic properties of Cr(VI) have long been known [6]. The genotoxic effects of Cr(VI) are associated with its ability to pass through cell walls and be reduced in the cell to Cr(III). Cr(III) is likely bound to phosphate DNA, inducing changes in the genetic code of the cell. The transitive species of Cr(V) and Cr(IV) and organic radicals are also formed during the reduction processes [7]. Another risk, especially for workers working with cement or fresh concrete, is that Cr(VI) causes hypersensitive reactions and allergic eczema when in direct contact with human skin [8]. Eugeniusz [9] reported the concentration for an allergic reaction is 10 mg Cr(VI) per 1 kg of cement.

The presence of Cr(VI) compounds in cements is very dangerous in terms of their solubility and possible leaching of Cr(VI) in connection with the use of cement in concrete structures such as water tanks and pipes, which can lead to contamination of drinking water or chromium accumulation in the environment [10,11].

Cr(VI) leaching examinations play a vital role in assessing the environmental quality of cementitious materials. A variety of different leaching tests can be applied to cementitious materials to characterize their leaching behaviour, such as the equilibrium-based test EPA Method 1311: The Toxicity Characteristic Leaching Procedure (TCLP); kinetic-based Test ANS 16.1: Measurement of the Leachability of Solids; EPA Method 1312, Synthetic Precipitation Leaching Procedure (SPLP); or ASTM D5233-92 (ASTM Single Batch). Most of the national regulations for hazardous metals mention a simple extraction leaching test in which the pulverized sample is extracted with different aqueous solutions, usually acidic solutions. This is the case for TCLP, which uses an acetic acid solution with a liquid to solid ratio (L/S) of 20:1 for 18 h. Batch tests such as SPLP or TCLP may be adequate for investigating materials when the test is known to produce sufficient environmentally safe results [12]. However, the TCLP and ASTM Single Batch methods were reported in [13] as being inappropriate for chromium testing. Lackovic et al. [14], reported that the SPLP was more realistic than the TCLP for assessing the mobility of metals in soils; however, the SPLP underestimated the mobility of chromium.

Currently, the EU standard is applied for the determination of the water-soluble chromium (VI) content of cement, and this method is an extraction method similar to the TCLP that uses de-ionized water in a liquid to solid proportion (L/S) of 10:1 [15]. Another leaching protocol was adopted to determine the environmental quality of concrete [16].

Currently, the incorporation of various secondary materials (fly ash, slag, silica fume, and zeolite) in cement production is of interest to reduce cement production environmental impacts and improve the properties of cement composites. However, leaching of heavy metals from cement materials increases with a waste material addition [17]. The main objective of the paper was to perform a comparative study of ordinary Portland cement (OPC) and concrete composites with selected secondary materials, silica fume and zeolite, to examine Cr(VI) leaching. The aim was to investigate whether (a) a simple extraction leaching test in which the cement sample is extracted with deionised water is sufficient for the evaluation of the environmental safety of cementitious products, and whether (b) Cr(VI) leaching from cement materials increases with a secondary material addition.

## 2. Materials and Methods

### 2.1. Cements

Four samples of ordinary Portland cement (OPC1–OPC4), representing the CEM I cement type, from cement producers in the Slovak Republic were examined in the experiments. Cements from selected manufacturers can be considered geopolitically representative for OPC in the Central European region. All cement samples were provided by producers at the moment they were produced. The cement samples were used for the experiments without any modification.

### 2.2. Cement Composites

Three cement composite types, K1–K3, were investigated for Cr(VI) leaching. The mix proportions of the cement composites designed for the study are presented in Table 1. The K1 composite consisted of OPC (cement sample OPC1), water, aggregate (VSH, Geca, Slovakia), and a polycarboxylate-based plasticizer (Stachema, Bratislava, Slovakia). The K2 composite contained, in addition to the previously listed components, a silica fume addition (OFZ a.s., Istebne, Slovakia) representing 5.5% of the cement content. The chemical composition of the silica fume, measured by X-ray fluorescence spectrometry (XRF), in % by mass was as follows: 1.07 MgO, 0.36 Al_2_O_3_, 92.46 SiO_2_, 0.05 P_2_O_5_, 0.07 SO_3_, 0.14 Cl, 0.99 K_2_O, 0.23 CaO, 0.58 MnO, and 1.24 Fe_2_O_3_. The composition of the K3 composite differed from K2 by the addition of natural zeolite of 5.5% by cement mass. The main components of zeolite–clinoptilolite, which originated from regional sources, (Zeocem, Bystre, Slovakia) were as follows: MgO 1.18%, Al_2_O_3_ 14.14%, SiO_2_ 71.96%, P_2_O_5_ 0.05%, SO_3_ Sulfur 0.01%, Cl 0.004%, K_2_O 2.27%, CaO 3.16%, TiO_2_ 0.51%, MnO 0.03%, and Fe_2_O_3_ 3.61%. The mixtures were designed to possess workability comparable to that of the fresh concrete and the required durability and strength for the hardened concrete.

The aggregates, water, cement, and additives in designed amounts (Table 1) were thoroughly mixed, and the mixture was placed in forms with dimensions of 150 mm × 150 mm × 150 mm after the compacting process. The concrete specimens were placed in a water tank after unmoulding for 28 days. Curing helps to develop the properties of the concrete mixture so the concrete can develop the desired strength through a continued hydration process. After the curing period, all cement composites were cut into smaller specimens with dimensions of 50 mm × 50 mm × 10 mm for the leaching tests.

### 2.3. Leaching Experiments

Three different exposure environments, including deionized water, Britton-Robinson buffer (BRB), and hydrochloric acid (HCl), were simulated to investigate the leachability of hexavalent chromium from cement products under different environments. The laboratory experiments proceeded at room temperature (22–23 °C). Leaching of the cement samples consisted of two stages, an extraction procedure and an analysis of the filtered extract. The leaching time was dependent on the material studied. Cement samples were investigated by applying a short-term availability test, where the extraction time was not longer than 900 s [15]. After a longer extraction, the hydration processes began. The same extraction time of 900 s was used for all cements in all leachants to compare the results. A long leaching time was applied in the case of the monolithic composites in accordance with the tank test requirements in [16]. The pulp density was 10%, and a volume of liquid a minimum of ten-times higher was applied for the tank leaching, as recommended in [16].

To analyse the water-soluble chromium, 10 g of cement was mixed with 100 mL of deionized water (conductivity of 5.72 μS/cm and pH of 6.81) prepared by a reverse osmosis water system (Rodem 6, Intertek, Slovakia) for 900 s at the laboratory temperature. After extraction, the solution was filtered using the Morton bacteria vacuum filter system (Pyrex^®^) with a diameter of 40 mm and an ultra-fine porosity (0.9 to 1.4 µm). The obtained filtrate was diluted by deionized water to bring the volume up to 250 mL and analysed for the Cr(VI) content.

To simulate the natural environmental conditions, a Britton-Robinson buffer (BRB) was prepared by mixing the same volumes of 0.04 M H_3_BO_3_, 0.04 M H_3_PO_4_, and 0.04 M CH_3_COOH and subsequently titrating the mixture with 0.5 M NaOH to obtain a pH of 6.95. Ten grams of cement was treated with 100 mL of Britton-Robinson buffer for 900 s. After filtration with the Morton bacteria vacuum filter system, the obtained filtrate was analysed for the Cr(VI) amount.

The 10 wt. % HCl was used to dissolve the available chromium content in cements, and it was used to simulate an aggressive acidic environment. Approximately 10 g of cement was accurately measured and treated with 100 mL of 10 wt. % HCl for 900 s. Then, 0.5 M NaOH was used to adjust the pH value to neutral. The Fe(OH)_3_ that precipitated in solution during titration was removed by filtration [18].

To study the Cr(VI) leaching as a function of time, the cement samples were tested once a month over a period of 700 days. The cement material was kept under optimal conditions (a constant temperature between 20–22 °C, average relative moisture of 38%, and limited air access) during the experiment.

The leaching of the monolithic cement composites was studied by performing a continuous, 90-day, tank leaching test. This extraction procedure represents dynamic testing and is applied to evaluate metal release from porous materials when leaching is diffusion-controlled [19]. The leaching tests were carried out in sealed glass beakers using deionized water and 10 wt. % HCl as the leachants. After the period indicated, the leachate was filtered through a 0.4 mm glass membrane filter and acidified with HNO_3_. A fixed volume of 20 mL of each sample was analysed by a spectrophotometric method.

### 2.4. Analytical Methods

The chemical compositions of the cements and cement composites were investigated using X-ray fluorescence spectrometry, XRF SPECTRO iQ II (Ametek, Germany), with a silicon drift detector (SDD) with a resolution of 145 eV at 10,000 pulses. XRF was used for a wide range of elements at detection limits at the sub-ppm level.

The dissolved Cr(VI) amounts in the leachates were measured by a spectrophotometric analysis (DR 2800, Hach Lange, Germany) at a wavelength of 540 nm. The concentrations of soluble chromium were determined by the Diphenylcarbohydrazide Hach Method 8023 with Cr(VI) detection limit of 0.01 mg/L.

X-ray photoelectron spectroscopy (XPS) measurements were carried out on the SPECS electron spectrometer at a base pressure in the analysis chamber of 5 × 10^−10^ mbar (2 × 10^−8^ mbar during the measurements) using the anode MgKα and a non-monochromated X-ray source with an excitation energy of 1253.6 eV. Most elements are detectable at about the 1 at. % to 0.1 at. % level, for heavy elements (e.g., Cr) in a light element matrix the detection limit was 0.01 at. %. The spectra were recorded at the total instrumental resolution (as measured with the FWHM of the Ag 3d^5/2^ photoelectron line) of 1.06 eV for the MgKα excitation source. The data were analysed using CasaXPS software (Casa Software Ltd., Teignmouth, United Kingdom). The energy scale was calibrated by normalizing the C 1 s line of the hydrocarbons to 285.0 eV. The processing of the measured spectra included a subtraction of the X-ray satellite peaks and Shirley-type background. The peak positions were evaluated by a symmetrical Gaussian-Lorentzian curve fitting. The relative concentrations of different chemical species were determined based on a normalization of the peak areas to their photoionization cross-sections calculated by Scofield. The XPS of all spectra were recorded to obtain information on the chromium species in the samples.

## 3. Results and Discussion

### 3.1. Chemical Compositions of the OPCs and Cement Composites

The results of the XRF chemical analysis of the OPCs and cement composites are shown in Table 2.

The basic chemical composition of the studied cements correlates with the standard composition of OPC containing only clinker and no other single constituents [20,21]. The chemical compositions of the analysed OPCs and cement composites were similar and varied with only calcium and silicon contents. OPCs contained lower amounts of SiO_2_ up to 19%, while the SiO_2_ content in cement composites was up to 45.6%. The higher SiO_2_ content in the composites was associated with aggregate, zeolite, and silica fume addition (samples K2 and K3). The content of CaO in the cement samples was measured up to 63.9%, while it was in the range of 26.2–31.3% in the composites.

The total chromium concentrations in the analysed OPC cement samples measured by XRF ranged from 173.2–218.5 mg/kg (Table 2), which correlated with the total chromium concentrations in the cements from the Czech producer, that is, 87 to 283 mg/kg [22]. Frias and Rojas [23] reported the total content of all chromium species in cements as 20–110 mg/kg, whereas Lu et al. [24] measured an extremely low content of 17.1 mg/kg. The data from producers [25] in the central European region indicate that the raw material itself contributes up to 30–40 mg/kg of the total chromium concentration in clinker. This concentration can be increased by iron corrections.

The total chromium content in the composites was higher than that in the OPCs and ranged from 180.4 to 485.0 mg/kg.

### 3.2. Influence of the Leaching Agent on Hexavalent Chromium Leaching

The results indicated that the leachable portion of chromium is strongly dependent on the ambient environment. Generally, chromium in cements was leachable in descending order of HCl, BRB, and deionized water.

#### 3.2.1. Deionized Water

The results of the water-soluble Cr(VI) monitoring in cement samples and cement composites are presented in Table 3.

The amount of leached chromium ranged from 0.23 to 3.19 mg/kg with an average value of 2.1 for the OPCs. The most intensive leaching of Cr(VI) was observed for the OPC1 sample. The measured values were lower than those measured by other authors in the past [5,18,26]. The concentrations of hexavalent chromium in Japanese [18] and Australian cements [26] were measured to be in the range of 0.2–20 mg/kg. Frias and Rojas [23] reported the water-soluble hexavalent fraction was 0.9–24 mg/kg. The lower water-soluble hexavalent chromium quantities in our study could be linked to more stringent legislative measures; in May 2003, the European Council adopted Directive 76/769/EEC [27], which prohibits packaged cement with a Cr(VI) content higher than 2 mg/kg to be placed on the markets in EU countries.

Several authors have studied Cr(VI) occurrence in OPC [28,29,30,31]. Chromium leaching depends on various factors [23] but, according to Bentaieb et al. [32], it is strongly linked to the chemical composition of the cement clinker. A substantial reduction in the Cr(VI) chromium content in cements to below the limit of 2 mg/kg can be achieved by a primary reduction of the total chromium in the clinker related to the raw materials and by replacing ferrite ingredients. The reference document for the production of cement [33] stated that the total chromium content ranged from 10–40, 1.2–21, and 20–109 mg/kg in the raw materials, limestone, and clay minerals, respectively. Sinyoung et al. [34] reported that chromium could either be incorporated in clinker phases or form a new crystalline phase during a reaction at high temperatures with several oxidation states. As mentioned by Strigac et al. [25], in the cement clinker, there is still a high occurrence of Cr(VI) ranging from 6–14 mg/kg according to the location. In addition, when sulfate-based regulators (CaSO_4_) are added to the clinker, they can increase the overall hexavalent chromium quantities in OPCs by promoting the solubility of CrO_4_^2−^ in aquatic environments. To achieve a reduction in Cr(VI) below the level of 2 mg/kg, the current manufacturing process requires adding reducing agents (soluble salts of Fe(II) and slag sulfides) to the cements, which reduce Cr(VI) to Cr(III) according to the following reaction (Equation (1)).

Cr^VI^ + 3Fe^II^ ⟶ Cr^III^ + 3Fe^III^ (1)

Based on the testing, it can be assumed that the reducing agents have been used in the studied OPCs to meet the EU requirements.

The Cr(VI) leached-out quantities versus the total chromium concentrations ranged from 0.08 to 1.79%. That means that the maximum amount of water-soluble Cr(VI) in the OPCs represented only 1.8% of the total chromium amount available for leaching. This prompted an interest in testing another aqueous leaching agent to confirm or reject the relatively low Cr(VI) leachability.

For the composites, the Cr(VI) leached amounts were measured periodically over a period of 90 days, and the amounts varied in the range 0.39–1.41 mg/kg with an average value of 0.92 mg/kg (Table 3). Lu et al. reported that the amount of Cr leached from a concrete block after 64 days due to simulated sea water was 0.17 mg/kg, but the leached amount increased remarkably up to 6.1 mg/kg when the particle size of the cement mortar was reduced to 1.0 mm [24]. The results of a number of studies have demonstrated that the leachability of metals is intimately associated with the ambient environment and particle size of the cement mortar [35].

The quantities of chromium leached from the composites with secondary materials (K2 and K3) reached higher values than those from the OPC-based composites (K1) (Table 3). The highest mean concentration of water-soluble Cr(VI) was found for the K3 sample, including both silica fume and zeolite secondary materials, based on the highest chromium content. This finding indicates that the leaching of concrete blocks with secondary materials could be more significant in terms of the water-soluble Cr(VI), as assumed.

When comparing the percentage of water-soluble Cr(VI) quantities measured in particular cement composites and OPCs, it was found that more chromium leached out of cements than monolithic composites. This was also valid in the case of Cr(VI) leaching from powdered composites.

#### 3.2.2. BRB

**Figure 1:**
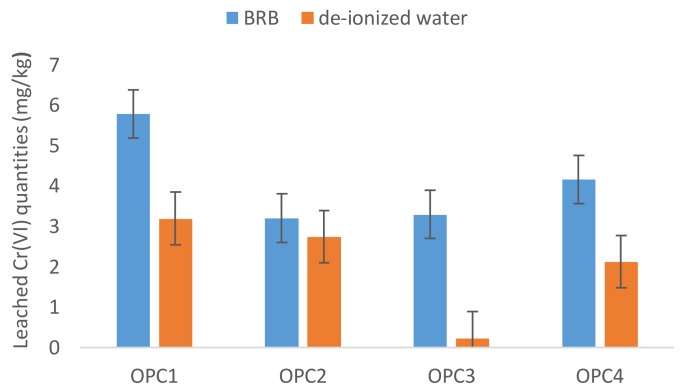
A comparison of the Cr(VI) leached amounts from cements in deionized water and Britton-Robinson Buffer (BRB).

To confirm or refute the relatively low Cr(VI) leachability, a Britton-Robinson buffer (BRB) with a pH of 6.95, which simulates a natural environment, was used for the leaching tests of the cement samples. The Cr(VI) amounts extracted by BRB were measured in the interval 0.55–5.78 mg/kg. The results obtained, as reported in Figure 1, showed that the water-soluble Cr(VI) values were markedly less than those obtained with the BRB leaching method, although the pH of BRB was slightly higher.

**Figure 2:**
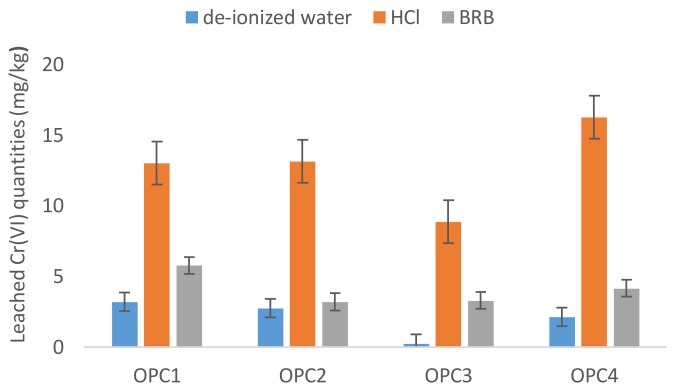
A comparison of the Cr(VI) amounts leached from cements in deionized water, BRB and HCl.

As shown in Figure 2, the average dissolved Cr(VI) amounts in BRB exceeded the dissolved amounts in deionized water by several fold, that is, 1.05 to 14.3 times. The Cr(VI) leached quantities in BRB represented 0.22–3.24% of the total chromium in the OPCs. The maximum percentage of Cr(VI) dissolved in the BRB leaching tests was higher than that in deionized water as well. This finding indicates that the standard leaching tests in only deionized water might not be accurate for leaching estimations under natural conditions.

#### 3.2.3. HCl

Considering the pH, the minimum values of leached chromium were observed in the pH interval 6.5–7.5, as mentioned in [36]. In light of this behaviour of chromium, an HCl solution with a pH of 2.5 was used for leaching experiments. The results of the Cr(VI) leaching by HCl for both the cement samples and composites are presented in Table 4.

The amount of Cr(VI) leached from cements ranged from 8.88–16.25 mg/kg, which was higher for all cement samples than that extracted by both deionized water and BRB (Figure 2). The results of the leaching tests indicate that the water-soluble Cr(VI) in cements represents approximately 2–25% of the chromium leachable by HCl, which agrees with [30,37]. This fact is probably linked to the presence of other Cr(VI) compounds with limited solubility in water. In addition to Cr(VI) water-soluble compounds (Na_2_CrO_4_, K_2_CrO_4_ and MgCrO_4_), insoluble Cr(VI) salts, such as PbCrO_4_, BaCrO_4_, and ZnCrO_4_, have been confirmed to be present in cements.

The highest dissoluble Cr(VI) percentage, ranging from 1.46 to 9.45%, was found after chromium dissolution in HCl. However, the Cr(VI) dissolved in HCl cannot be considered as the total amount of hexavalent chromium in cements because hexavalent chromium can be reduced in the presence of HCl to trivalent species under specific conditions (Equation (2)).

2K_2_CrO_4_ + 16HCl ⇔ 4KCl + 2CrCl_3_ + 8H_2_O + 3Cl_2_ (2)

Thus, the overall content of Cr(VI) may be much higher than the leaching results indicated.

**Figure 3:**
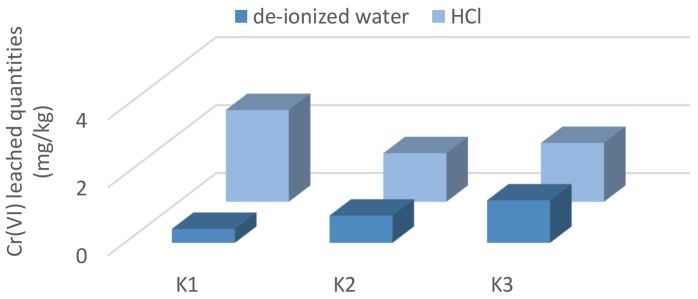
A comparison of the Cr(VI) leached quantities from composites in deionized water and HCl after the 90-day leaching.

Similarly, higher Cr(VI) leached quantities have been measured for monolithic concrete immersed in HCl (1.2–5.7 times) than those in deionized water. A comparison of the Cr(VI) dissolved amounts by deionized water and HCl after the 90-day experiment is illustrated in Figure 3.

Surprisingly, when comparing the average dissolved Cr(VI) and the percentage of the particular composite, the highest leachability in HCl was with the K1 composite without any secondary material addition.

### 3.3. Chromium Species in Cements

**Figure 4:**
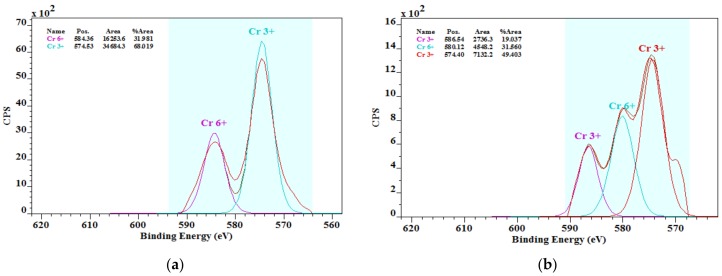

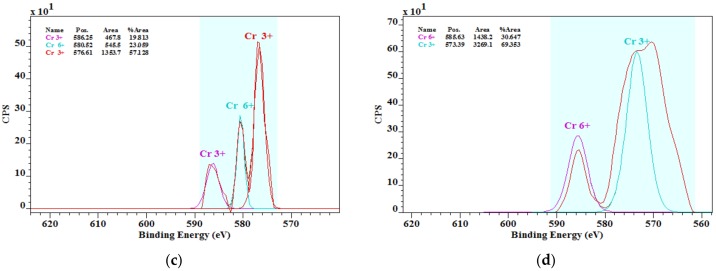
X-ray photoelectron spectroscopy (XPS) spectra of (**a**) OPC1; (**b**) OPC2; (**c**) OPC3; and (**d**) OPC4.

According to literary knowledge, chromium in cement is found in trivalent and hexavalent forms, and authors have indicated a broad range of Cr(VI) in cements [4,5]. Therefore, investigations on metal speciation and leaching behaviour are important to determine the risks to the environment and human health. Yamaguci et al. [18] showed that between 30 and 80% of the total chromium in cement clinkers is represented by Cr(VI) compounds. For the purpose of a more detailed study of the oxidation states of chromium and their percentages in our OPCs, an X-ray spectroscopic analysis (XPS) was used. The XPS method is suitable for analysing all solid materials when assuming homogeneity throughout the volume, including bulk materials. Figure 4 presents the XPS spectra of the analysed OPC samples.

In addition to the peaks corresponding to the alkali metal and alkaline earth metal chromates, the presence of dichromium tris(chromate), Cr_2_(CrO_4_)_3_, with a bonding energy of 579.7 eV was observed. This compound represents the occurrence of both chromium species in one chemical compound. The presence of Cr_2_(CrO_4_)_3_ may be related to the differences in the assumed solubility of the overall hexavalent chromium content in the aqueous medium.

In all the analysed samples of cement, the XPS analysis confirmed the presence of chromium in both oxidation states, that is, VI and III (Table 5), with the trivalent species consisting of up to 77%, as seen in Table 5. The predominance of the trivalent species is in contrast with the results of Lee et al. [38], who reported that the chromium in OPCs is mostly in the form of chromates.

Considering the XPS results, the hexavalent chromium content in the studied OPC samples represented only 23 to 32% of the total chromium content. Based on this finding, it is possible to determine that the overall Cr(VI) content in the analysed cements is in the range of 30.8–66.1 mg/kg. From the overall content of Cr(VI) in cements, only a maximum of 10.4% of hexavalent chromium was soluble in the aqueous medium. Potgieter et al. [4] investigated OPCs and reported more Cr(VI) species were soluble in water, that is, approximately 15%. The general perception that all hexavalent chromium in cement and cement-related materials is water soluble has not been proven conclusively, as mentioned in [4].

### 3.4. Dependence of Hexavalent Chromium Leaching on the Cement Age

**Figure 5:**
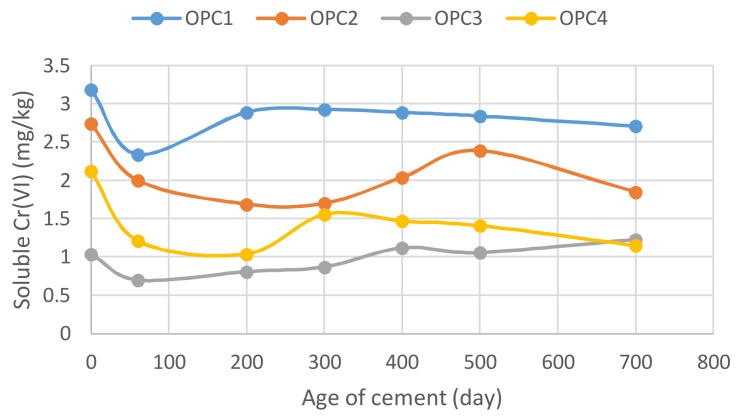
Cement age influence on Cr(VI) quantities leached from OPCs.

It is generally known that cement should not be stored for more than 3 months even under the recommended storage conditions, i.e., original, intact, dry package and low humidity. During long-term storage, cement can react with air humidity and carbon dioxide, resulting in partial hydration and carbonization. This reflects a decrease in the strength of the stored cement. After three months, the strength of even properly stored cement is reduced by approximately 10 to 20%, and after six months by 20 to 30%. In addition, the storage of cement may also result in a reduction in the effect of the reducing agents that reduce the soluble chromium content below the required ≤0.0002% of dry cement by mass. The reducing agent is effective until the date of expiration indicated on the packaging. After that time, the agent can lose its effectiveness, and the Cr(VI) content may increase. In accordance with this knowledge, the soluble Cr(VI) amount was supposed to increase during the storage of OPCs. However, as seen in Figure 5, a significant decrease in the water-soluble Cr(VI) concentrations was observed for all OPC samples on the 60th day of cement storage. A concentration minimum was observed for all investigated samples; thus, there is a decreasing trend at approximately 60 to 200 days.

This decrease could be connected with the partial hydration processes taking place during the long-term, 60-day or more storage, since chromium can enter the belite structure during hydration and support and accelerate hydration reactions [22,39]. After that, the water-soluble Cr(VI) concentrations in the OPCs tended to increase again, which was probably due to the loss of the effectiveness of the Cr(VI) reducing agents and oxidation processes during cement aging, as reported in [40]. The water-soluble Cr(VI) concentrations in the OPC samples varied from 0.7 to 3.2 mg/kg during the 700 days of testing. Comparing both the original and final concentrations, it can be concluded that the Cr(VI) concentrations slightly decreased after the 700-day cement storage (Figure 5). This finding can likely be explained by the formation of another Cr(VI) compound with a lower solubility in water, since a reduction of the hexavalent form to a trivalent species of chromium is not presumed.

## 4. Conclusions

This study dealt with Cr(VI) leaching from OPCs and cement composites incorporating silica fume and zeolite. Deionized water, BRB, and HCl were used as the leaching media. The findings revealed the following:

- The OPCs average value of Cr(VI) dissolved in deionized water was lower than those reported in the literature in the past, which indicated that most manufacturers use reduction agents in the cement manufacturing process now. In spite of that, the EU limit value of 2 mg/kg for water-soluble Cr(VI) was exceeded in 27% of the analysed OPCs.
- The current recommended procedures for testing water-soluble chromium using extraction by deionized water seem not to predict the real portion of Cr(VI) dissolved from cement materials into the environment.
- The literature knowledge that the chromium in cements is mostly in the chromate form was not confirmed. In addition, the presence of chromium chromate Cr_2_(CrO_4_)_3_ was found by the XPS analysis. More studies in this field on chromium species and their solubility are important.
- The assumption of the lower Cr(VI) leaching rate of monolithic concrete blocks compared to that of the OPCs was confirmed. The increased leaching in deionized water of chromium from cement composites with silica fume and zeolite was confirmed. Cementitious materials made of OPC and secondary materials show very systematic leaching behaviours.
- The prediction of leachability from the tank leaching test could be a basis for geochemical modelling.
- A minimum concentration was observed for all cement samples when studying the relationship between the soluble Cr(VI) and cement storage time.

The results of the study show that some progress has been made in the production of cements in the EU, with respect to the content of hexavalent chromium compared to the results of authors in the past. However, because of the seriousness of the negative impacts of chromium on human health, the content of hexavalent chromium in cement materials still needs to be monitored. Likewise, instead of monitoring the relatively low leaching rate of hexavalent chromium from concretes, the environmental safety of concrete should be based on analyses of waste and secondary materials with precise testing methods.

## Figures and Tables

**Table 1:** Cement composite mixtures—composition per 1 m^3^ of fresh concrete.

Cement Composite	Components Per 1 m^3^ of Cement Composite							
	Cement (kg)	Water (L)	Silica Fume (kg)	Zeolite (kg)	Aggregate (kg)			Plasticizer (L)
					0/4 mm	4/8 mm	8/16 mm	
K1	360	170	-	-	825	235	740	3.1
K2	360	197	20	-	800	235	740	3.1
K3	360	205	20	20	750	235	740	3.1

**Table 2:** Portland cement and composite components.

Sample	Elements in Oxide Form (wt. %)									Cr (Total) (mg/kg)
	CaO	SiO_2_	Al_2_O_3_	Fe_2_O_3_	SO_3_	MgO	K_2_O	TiO_2_	MnO	
OPC1	58.2	19.6	4.4	3.3	3.2	3.8	0.6	0.2	0.4	178.5
OPC2	54.2	17.8	4.1	2.6	3.3	1.5	1.2	0.2	0.3	173.2
OPC3	63.6	19.8	3.9	2.7	3.1	2.1	0.5	0.2	0.3	210.1
OPC4	63.9	19.2	4.2	2.4	3.2	2.0	0.7	0.2	0.3	218.5
K1	31.3	30.2	5.2	4.0	2.9	3.0	0.8	0.3	0.4	180.4
K2	26.2	45.6	5.4	3.4	2.7	2.7	0.8	0.3	0.4	233.5
K3	25.1	39.8	5.3	4.6	2.8	2.4	0.8	0.3	0.4	485.0

**Table 3:** The concentrations of water-soluble Cr(VI).

Sample	Cr (VI) Concentrations (mg/kg)					Cr (VI)/Cr (Total) (%)
	Number of Leachates	Min	Max	Mean	Standard Deviation	
OPCs	12	0.23	3.19	2.10	0.87	0.8–1.79
Composites	12					
K1	4	0.39	0.55	0.41	0.08	0.26
K2	4	0.80	1.04	0.85	0.18	0.36
K3	4	1.25	1.44	1.36	0.25	0.28

**Table 4:** Cr(VI) amounts dissolved by HCl.

Sample	Cr(VI) Concentrations (mg/kg)					Cr(VI)/Cr (Total) (%)
	Number of Leachates	Min	Max	Mean	Standard Deviation	
OPCs	12	8.88	16.25	12.8	7.58	1.46–9.45
Composites	12					
K1	4	2.10	4.47	2.70	2.02	1.50
K2	4	1.10	3.51	1.43	1.08	0.61
K3	4	1.43	2.45	1.73	0.90	0.36

**Table 5:** XPS semi-quantitative analysis of the cement samples.

Sample	Concentration of Chromium Species (%)	
	Cr(III)	Cr(VI)
OPC1	68.09	31.91
OPC2	68.44	31.56
OPC3	76.94	23.06
OPC4	69.35	30.65
